# Semantic Quantum Communication: Merging Semantic Extraction with Quantum Security for Efficient Secure Transmission

**DOI:** 10.34133/research.1397

**Published:** 2026-08-28

**Authors:** Min Wang, Gui-Fa Zhu, Guo-Fei Long, Jianxing Guo, Yu-Chen Liu, Dong Pan, Athanasios V. Vasilakos, Li-Ping Nong, Gui-Lu Long

**Affiliations:** ^1^ Beijing Academy of Quantum Information Sciences, Beijing 100193, China.; ^2^ College of Physics and Technology, Guangxi Normal University, Guilin 541004, China.; ^3^ Beijing TGW Quantum Technology Co., Ltd., Beijing 100015, China.; ^4^ Department of Physics, State Key Laboratory of Low-Dimensional Quantum Physics, Tsinghua University, Beijing 100084, China.; ^5^ Center for AI Research (CAIR), University of Agder (UiA), Grimstad, Norway.; ^6^ School of Information and Communication, Guilin University of Electronic Technology, Guilin 541004, China.; ^7^ Frontier Science Center for Quantum Information, Beijing 100084, China.

## Abstract

Quantum secure direct communication (QSDC), a paradigm-shifting breakthrough in quantum communication, exploits quantum states for unmediated information transmission. Rooted in the inviolable fundamental laws of quantum mechanics, QSDC enables ultrasensitive detection of even the faintest eavesdropping attempts, guaranteeing true communication security solely when no interference exists. However, its practical scalability remains constrained by insufficient transmission rates. Semantic communication, which drastically boosts transmission efficiency by extracting core information features, nevertheless stays vulnerable to malicious intrusions. Integrating these paradigms promises to simultaneously enhance the equivalent data rate and security. Herein, we propose and experimentally validate a quantum semantic communication scheme, applying it to 3-dimensional point clouds. It achieves a 46.30-fold efficiency gain over direct transmission in the meaning domain, beyond the Wyner and Shannon capacity limits of syntactic communication. This breakthrough not only broadens the application scope of QSDC with limited bandwidth, but also marks a pivotal milestone in quantum information science.

## Introduction

Quantum communication [1–6] and semantic communication [7–9] are the twin pillars of the new paradigm reshaping communication science, with quantum secure direct communication (QSDC) [3,10] and semantic data compression as core drivers. QSDC redefines secure data transfer as it can detect the slightest eavesdropping attempts, alert users to potential exposure, prevent unauthorized tracking, and neutralize hacker threats. Semantic communication boosts transmission efficiency through semantic compression, extracting and retaining only key informational features and discarding redundant data. It has high security requirements. Integrating quantum teleportation and remote state preparation paradigms with semantic compression holds the promise of revolutionizing quantum information extraction and transmission efficiency [11–15]. However, this approach is still a future prospect. In contrast, the semantic QSDC scheme [16], which combines semantic database construction with QSDC for secure transmission, makes a transformative leap in both transmission efficiency and security. With the rapid development of QSDC [17–30], it can be fully implemented using current technologies. Here, we propose and experimentally demonstrate the world’s first semantic QSDC scheme for 3-dimensional (3D) point clouds. This work enhances efficiency by 46.30 times compared to direct transmission in the meaning domain, beyond the Wyner and Shannon capacity limits of syntactic communication [31,32]. This work broadens the application scope of QSDC with limited bandwidth, marking a landmark milestone in quantum information science.

## Results

### The semantic QSDC framework

**Fig. 1.**
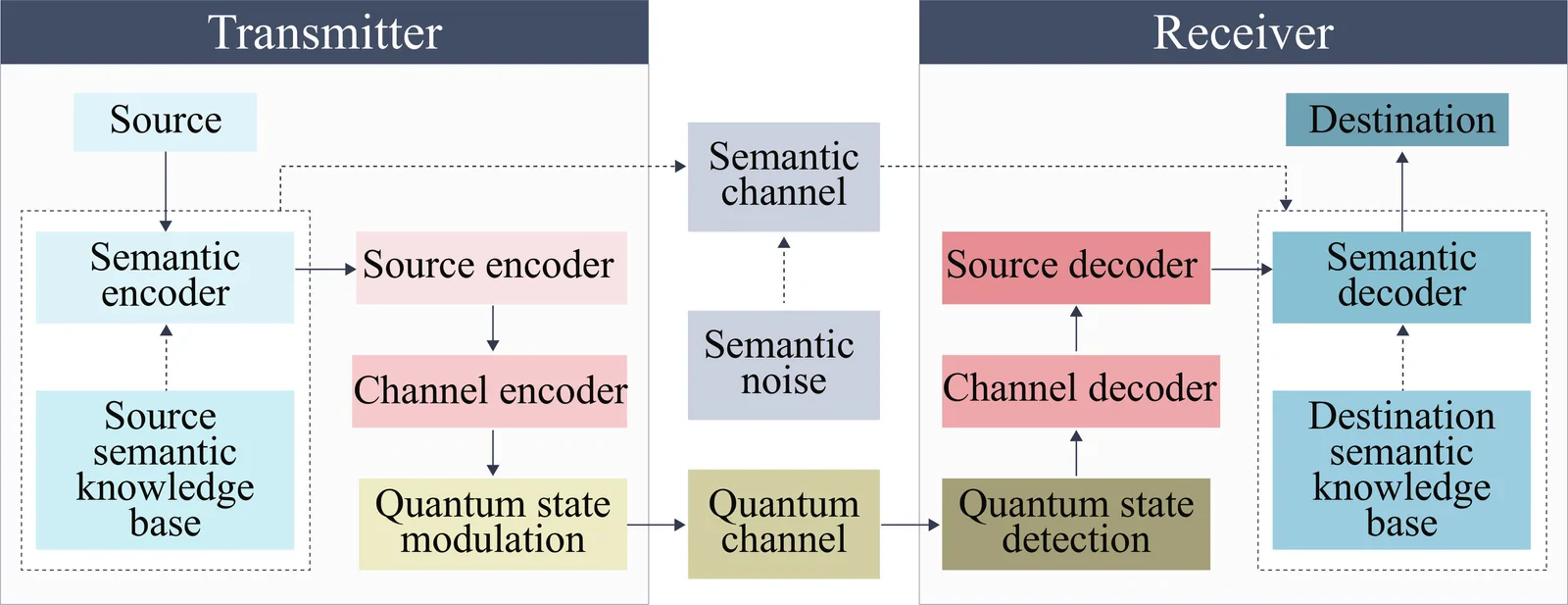
General framework for semantic quantum secure direct communication.

In semantic QSDC, the communication chain consists of a transmitter, a quantum channel, and a receiver (Fig. 1). The process starts with the information source generating raw data (e.g., point clouds, speech, and text). At the transmitter, the semantic encoder processes the input data using the source semantic knowledge base, which offers key semantic priors and contextual insights, allowing the encoder to extract a compact and meaningful semantic representation.

Once the semantic representation is derived, the source encoder converts it into codewords or symbol sequences optimized for digital processing and transmission, reducing redundant data and ensuring format uniformity. Then, the channel encoder embeds redundancy into these symbol sequences to enhance error resilience. After that, the sequences are mapped to quantum states and transmitted via QSDC technology. Importantly, before transmitting these encoded quantum states, the quantum channel is preauthenticated [33,34], providing a secure basis for the whole communication process.

During transmission, the quantum channel suffers from physical-layer noise that distorts transmitted quantum states. Meanwhile, the communication process is affected by the semantic channel, which models semantic-level distortions. In the context of source and destination semantic knowledge bases, these distortions, called semantic noise, may involve random erasure of semantic content, incorrect substitution of semantic features, or overall semantic blurring and drift. Semantic noise usually results from contextual mismatches, inconsistencies between knowledge bases, or inference errors, making it difficult for the receiver to accurately recover the intended semantics. The semantic knowledge base is an inherent component of the semantic communication paradigm: it provides the contextual priors that enable efficient compression and robust reconstruction [35]. In our framework, the semantic knowledge base is specifically designed for 3D point cloud data; extension to other data modalities would require constructing domain-specific semantic bases adapted to the characteristics of the corresponding data type.

At the receiver, incoming quantum states are detected and measured to get symbol sequences. The channel decoder uses error correction and recovery algorithms to reconstruct symbols close to the transmitted ones. The source decoder converts the recovered symbols back to the semantic representation. With the destination semantic knowledge base’s support, the semantic decoder interprets and reconstructs the semantics. The knowledge base offers contextual priors and structural information, enabling the decoder to compensate for missing elements due to semantic noise, resolve ambiguities, and restore an output similar to the original data. Finally, the system generates decoded content for humans or higher-level tasks, and a feedback mechanism can return confidence levels or error information to the transmitter to update the source and destination knowledge bases and optimize transmission performance.

3D point clouds [36–38], an essential data format for capturing spatial geometric structures, have become a core modality for future information interaction and intelligent perception. However, because of high dimensionality and sparse distribution, quantum communication for point clouds is currently impracticable because of the current technological limits, though QSDC offers high-grade security. In contrast, classical semantic communication has advanced in communication efficiency and robustness for 3D point clouds and shows promise in multimodal data transmission, like for images [39], speech [40], and text [41]. Deep learning models [42] have been proposed to extract essential features from point clouds, enabling effective reconstruction and recognition with minimal data transmission and reducing bandwidth constraints. Nevertheless, classical communication channels in this approach are still vulnerable to eavesdropping and attacks [43].

Combining point cloud semantic communication with QSDC, we construct a semantic QSDC of point clouds (S-QSDC-PC) scheme. It uses quantum communication’s inherent security for secure semantic data transmission and semantic encoding/decoding for efficient secure communication. This integration is beneficial in scenarios with high security and efficiency demands, like autonomous driving, smart cities, and military communications.

### Experimental results

We used the ShapeNet [44] dataset in our experiments. The dataset contains 51,127 point cloud samples across 55 common object categories, such as airplanes, chairs, and beds. It was divided into 35,708 training samples, 5,158 validation samples, and 10,261 test samples. Each point cloud was sampled to 2,048 points using the farthest point sampling (FPS) algorithm, and each point includes 3D coordinate information. The compression rate is determined by the length of the semantic code *n*. For example, when *n* = 50, the compression rate is $50/\left(2,048\times 3\right)\times 100\%=0.81\%$. In this study, *n* was set to $\left[10,20,50,100,200,300\right]$, and a separate model was trained for each case. Note that *n* = 6,144 represents the length of the raw point cloud when no semantic encoding is applied. Each model was trained for 200 epochs with a batch size of 32 using the Adam optimizer, with an initial learning rate of 0.001 that was halved every 20 epochs. After training, we used the models to encode and transmit test set point clouds. For each *n*, the corresponding model was evaluated, and the batch size was adjusted during transmission to match the bandwidth of the quantum channel.

**Fig. 2.**
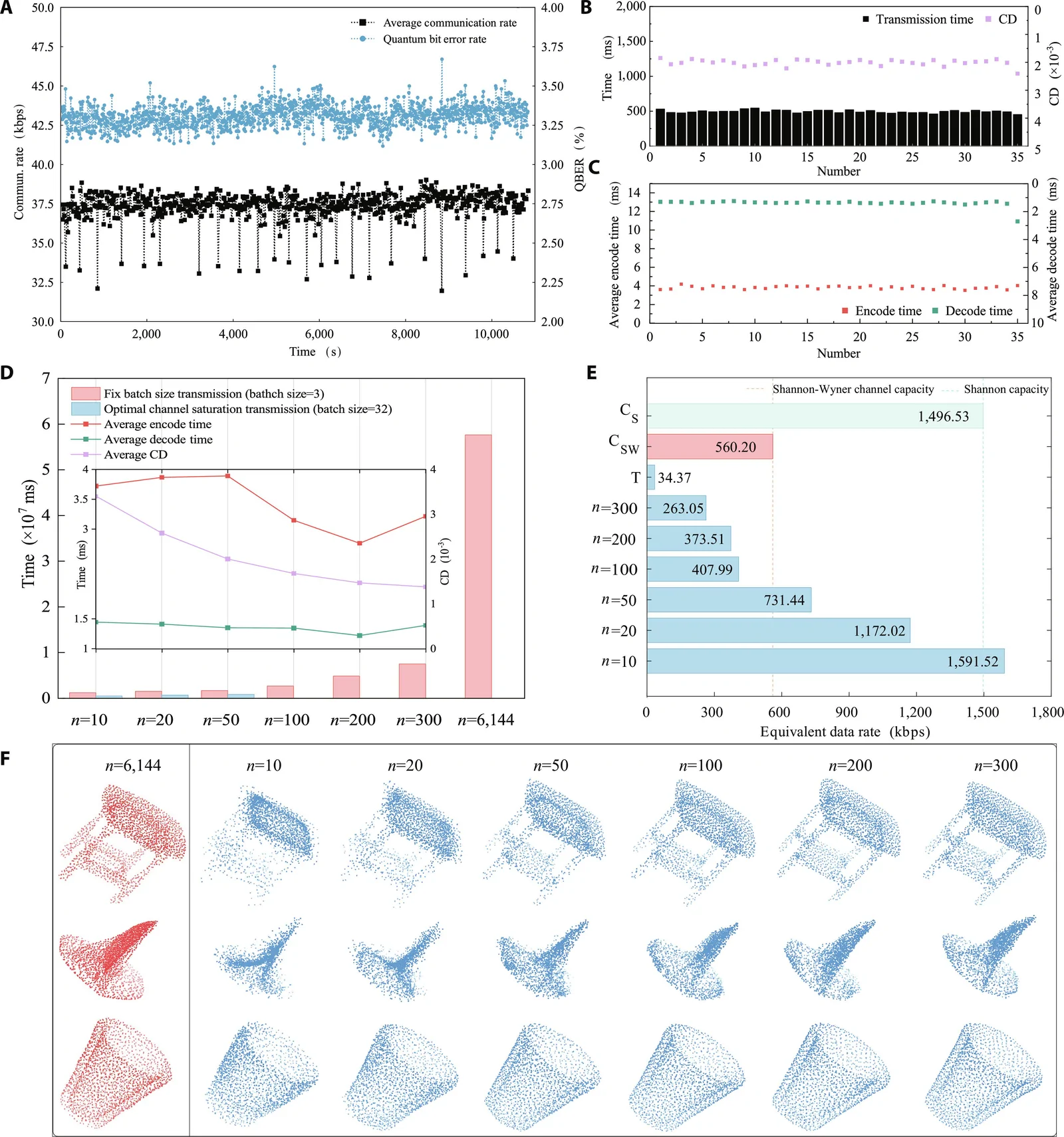
(A) Communication rate and QBER versus time of the QSDC system transmitting raw bits over a 50-km fiber link during 3 h. (B) Transmission time and CD under different numbers when *n* = 50. Each number represents 100 transmission rounds. In each round, 3 point clouds are selected from the dataset, encoded into a 3×n semantic feature, and transmitted once. The transmission time is measured from the start of encoding to the completion of decoding. (C) Average encode time and average decode time under different numbers when *n* = 50. (D) Comparison of transmission time, encode and decode, and CD under different *n* and batch sizes. Transmission time denotes the total time to complete all transmission tasks corresponding to each *n*. For batch size 3, all *n* are tested; for batch size 32, only n=10,20,50 are tested. In each transmission, a batch of point clouds is transmitted, and the process is repeated 10,261/batch size times. Encode and decode times and CD values are computed for n=10,20,50,100,200,300. Note that *n* = 6,144 corresponds to transmitting the original point clouds without encoding. (E) Transmission efficiency for different *n* with the Shannon–Wyner channel capacity as reference. At a fiber distance of 50 km, the first dashed line at 560.20 kbps corresponds to the secrecy capacity of the QSDC system derived from the Shannon–Wyner entropy, while the second dashed line at 1,496.53 kbps marks the mutual information between the quantum transmitter and quantum receiver, namely, the Shannon capacity. (F) Visualization of reconstructed point clouds for different *n*.

For the transport layer, the transmitter and receiver established a quantum channel via a 50-km standard single-mode fiber with an average link loss of 0.2 dB/km. The transmitter used weak coherent light as the light source with an operating wavelength of 1,550 nm, a repetition rate of 1.25 GHz, and a pulse width of 50 ps. The system employed 1 signal state and 2 decoy states, where the average photon number of the signal state was 0.6 and those of the decoy states were 0.2 and 0. To deal with loss and noise in the quantum channel, coding techniques like spread spectrum and low-density parity-check code were used, resulting in a spreading ratio of 1:192 and a forward error correction ratio of 1:12 in the experiment. The receiver used an InGaAs/InP single-photon detector with a detection efficiency of 20% and a dark count rate of $1.2\times 10^{-6}$ per gate. As shown in Fig. 2A, the average quantum bit error rate (QBER) of the quasi-QSDC system was 3.31%, well below the one-way quasi-QSDC protocol threshold. The average communication rate during 3 h of data collection was 37.36 kbps in the 50-km link, ensuring safe and smooth transmission of semantic codes.

*Transmission data analysis*: We first measured encoding time, decoding time, Chamfer distance (CD), and total transmission time at a fixed semantic code length of n=50. The batch size was set to 3, and reported values are averages per 100 samples. As shown in Fig. 2B and C, encoding time is about 4 ms, decoding time is about 1.5 ms, channel transmission time is around 500 ms, and CD fluctuates near $2\times 10^{-3}$. These results show that processing delays are negligible compared to channel latency, and the system has a relatively low and stable distortion level. This indicates that semantic communication at n=50 can effectively balance transmission efficiency and reconstruction quality, crucial for real-time applications under bandwidth constraints.

We then evaluated the total transmission time of the test set, including both encode and decode and channel transmission. First, we compared the total transmission time for different code lengths under a fixed batch size of 3 and a saturated channel with a batch size of 32. As shown in Fig. 2D, the saturated channel reduced the transmission time by 59.93%, 54.48%, and 47.51% for *n* = 10, *n* = 20, and *n* = 50, respectively, compared to the fixed batch size transmission. Furthermore, relative to the direct transmission of raw point clouds, the saturated channel reduced the transmission time by 91.34%, 87.88%, and 84.61% for *n* = 10, *n* = 20, and *n* = 50, respectively. These results clearly demonstrate the substantial improvement of semantic communication in transmission efficiency under limited bandwidth.

We investigated the impact of semantic code length on processing delays and reconstruction accuracy. With a batch size of 3, average encoding and decoding times were evaluated at different *n* values. The results in Fig. 2D show that the maximum encoding time was 3.89 ms at *n* = 50 and the maximum decoding time was 1.44 ms at *n* = 10. Both delays were at the millisecond level, meeting real-time communication requirements. Additionally, we analyzed the relationship between semantic code length and CD (a CD of 0 means the reconstructed point cloud is identical to the original). As *n* increased, CD decreased, indicating that larger code lengths help the model retain more geometric details and achieve higher reconstruction accuracy.

We summarize transmission results in Table 1 for intuitive comparison. We report average encode and decode latency, average CD values, and total transmission time under different semantic code lengths *n*. To evaluate performance, we introduce equivalent data rate (EDR) and relative transmission efficiency (RTE). EDR is the ratio of total bits of reconstructed point clouds to total transmission time at a given *n*, and RTE is the ratio of direct raw point cloud transmission time to that of tasks with code length *n*. Overall, we achieve an EDR of 34.37 kbps for raw point cloud transmission, and reach 1,591.52 kbps at n=10, 731.44 kbps at n=50, and 263.05 kbps at n=300. As shown in Fig. 2E, the EDR at n=50 exceeds the Shannon–Wyner channel capacity with a CD value of $2.00\times 10^{-3}$, and the EDR at n=10 exceeds the mutual information at the cost of data quality. In terms of RTE, the system improves efficiency by 46.30 times at n=10 compared to direct transmission. A balanced performance is obtained at n=50 with a 21.28-fold gain. Even at n=300 for higher visual quality, the system still improves by 7.65 times.

**Table 1.** Performance of the semantic quantum secure direct communication of point clouds

*n*	Time (ms)			CD	EDR	RTE
	Encode	Decode	Total	(×10^−3^)	(kbps)	
6,144	0	0	57,636,000	0	34.37	1
10	3.72	1.44	1,244,715	3.40	1,591.52	46.30
20	3.86	1.41	1,690,240	2.58	1,172.02	34.10
50	3.89	1.35	2,708,329	2.00	731.44	21.28
100	3.15	1.34	4,855,498	1.68	407.99	11.87
200	2.76	1.22	5,303,667	1.47	373.51	10.87
300	3.21	1.39	7,530,870	1.38	263.05	7.65

*Visualization of transmission results*: To further illustrate the effect of semantic code length on reconstruction quality, we visualized results for Chair, Cap, and Mug categories at different *n* values. As Fig. 2F shows, larger code lengths led to smoother surfaces, more uniform point distributions, and more accurate geometry in the 3 categories. This confirms that increasing semantic code length enhances reconstruction quality while maintaining transmission efficiency.

## Discussion

In this work, we propose a quantum semantic communication scheme. Semantic communication improves efficiency by extracting essential information and eliminating redundancy, while quantum communication safeguards transmitted semantic features against adversaries. We experimentally demonstrate semantic QSDC of point clouds. At n=10, a 46.30-fold efficiency improvement is achieved compared to direct point cloud transmission. Considering the trade-off between transmission quality and efficiency, at n=50, balanced performance is obtained. The EDR exceeds the Shannon–Wyner channel capacity with a CD value of $2.00\times 10^{-3}$, and a 21.28-fold efficiency gain is achieved. Even at n=300 for higher visual quality, the system still provides a 7.65-fold efficiency improvement.

To the best of our knowledge, this work represents the first experimental integration of QSDC and semantic communication for 3D point cloud transmission. Existing studies on quantum semantic communication mainly focus on theoretical analysis, while experimental validation for high-dimensional semantic data transmission remains largely unexplored. In contrast, the proposed framework establishes an end-to-end semantic communication architecture including semantic feature extraction, quantum-secure transmission, and semantic-guided point cloud reconstruction, and experimentally validates its feasibility over a 50-km optical fiber link.

The proposed framework also exhibits strong scalability within the semantic communication paradigm. Since only compact semantic representations are transmitted, the communication overhead mainly depends on semantic content complexity rather than raw data volume. Moreover, the framework can be extended to other semantic modalities, such as images, speech, and text, by redesigning the semantic encoder–decoder models and constructing domain-specific semantic knowledge bases.

Several important issues deserve further investigation in future work. First, extending the framework to additional data modalities requires retraining semantic models and constructing corresponding semantic knowledge bases. Second, the impact of practical quantum channel impairments, such as photon loss and detector noise, on semantic reconstruction fidelity remains insufficiently explored. Third, semantic communication emphasizes semantic fidelity rather than bit-level exact recovery, which may not satisfy applications requiring strict transmission accuracy. Future work may further investigate hybrid transmission frameworks integrating semantic communication with classical error correction under practical quantum channel impairments.

Despite its potential, quantum semantic communication faces challenges. Semantic communication models need further refinement to enhance generalization and reduce semantic noise in semantic knowledge-base construction. Quantum channels are prone to environmental interference, and limited transmission distances call for efficient quantum repeaters and error-correction schemes. As quantum memory, error correction, and semantic communication protocols mature, quantum semantic communication is expected to be a mainstream solution for smart cities, intelligent transportation, secure communications, etc.

## Methods

**Fig. 3.**
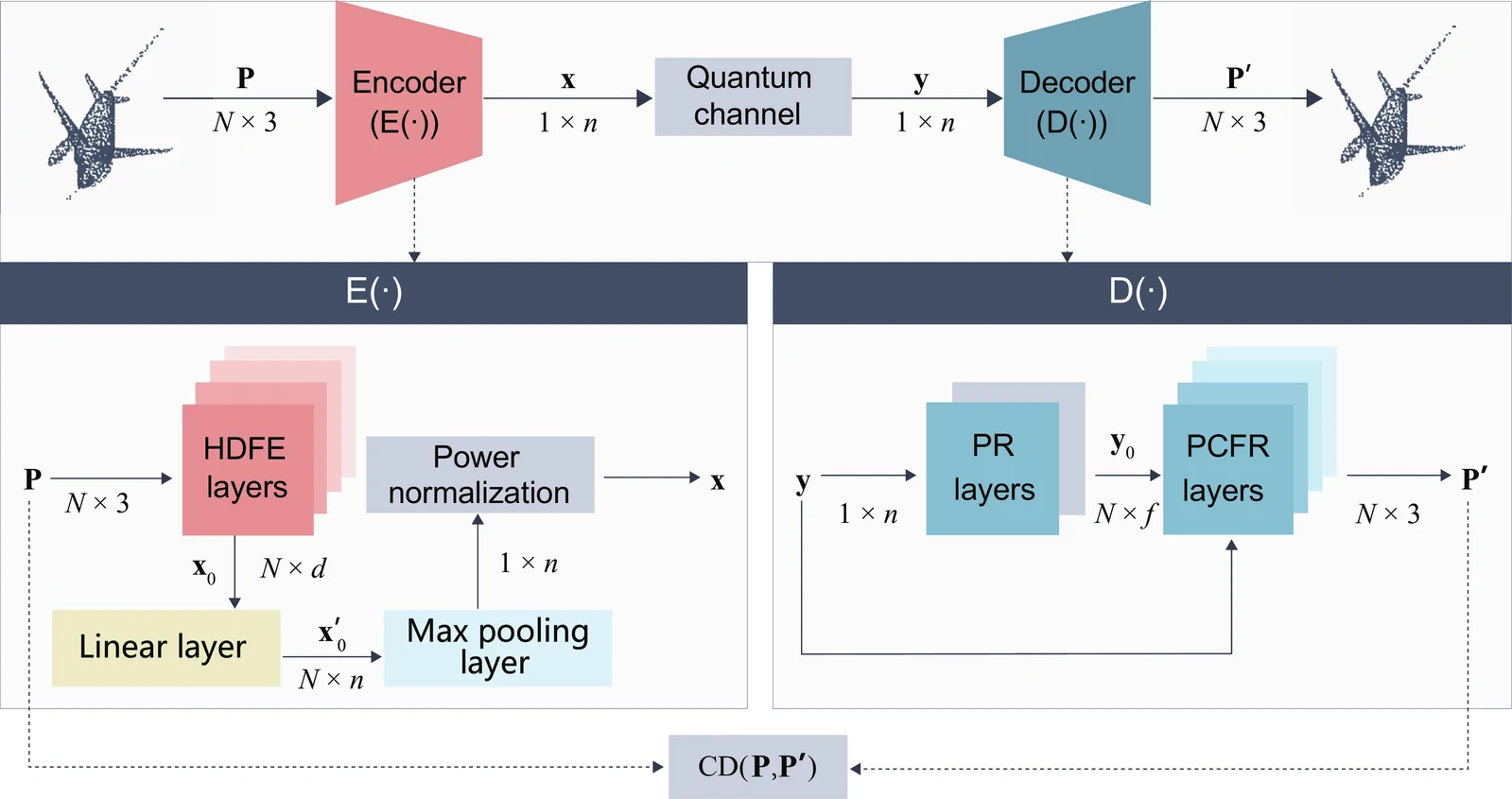
Framework of the semantic quantum secure direct communication of point clouds.

Our S-QSDC-PC system has 2 main components: an encoder and a decoder network, as seen in the $E\left(\cdot \right)$ and $D\left(\cdot \right)$ parts of Fig. 3. Specifically, we denote a single 3D point cloud as $P\in {\mathbb{R}}^{N\times 3}$, where *N* represents the number of points in this point cloud.

The encoder network takes a dense point cloud P as input and transforms it into point cloud semantic features $x\in {\mathbb{R}}^{1\times n}$, where *n* is the length of transmitted data, representing both the semantic code length and the raw data length in conventional transmission. To capture local geometric structures, a graph is constructed from the input point cloud based on interpoint distances using the *k*-nearest neighbors (*k*-NN) strategy [45]. The point cloud P and the graph’s adjacency matrix are input into 5 high-dimensional feature extraction (HDFE) layers based on graph convolutional networks [46]. Each HDFE layer maps point cloud features to higher-dimensional representations, leveraging the graph’s local neighborhood correlations to extract the point cloud’s semantic information. The HDFE encoder design is motivated by the inherently unordered nature of point cloud data: unlike images or sequential data, point clouds lack a fixed spatial ordering, which makes conventional grid-based or sequential encoders suboptimal. Graph convolution respects the natural structure of point clouds as spatial point sets, encoding 3D spatial relationships via dynamic local graph construction and enabling efficient feature propagation through message-passing mechanisms. The high-dimensional features $x_{0}\in {\mathbb{R}}^{N\times d}$ (where *d* is the number of feature channels from the last HDFE layer) are input to a linear layer. This layer maps $x_{0}$ to a low-dimensional $x_{0}^{\prime }\in {\mathbb{R}}^{N\times n}$. Then, a max-pooling layer selects the max value of each channel as the whole point cloud’s semantic information, changing the feature dimension from ${\mathbb{R}}^{N\times n}$ to ${\mathbb{R}}^{1\times n}$. Finally, a power normalization operation is used to get the point cloud semantic features x, which are sent through the quantum channel.

The semantic information of point cloud x is encoded into photon quantum states and transmitted via QSDC technology [10,27] over a noisy, high-loss channel to the receiver. We use the STIKE one-way quasi-QSDC protocol [25] for secure and reliable transmission. In this protocol, Alice encrypts information with preshared keys using a one-time pad and sends it via quantum states. Receiver Bob decrypts the information upon receipt and distills a key from the transmitted quantum states when the QBER is below the threshold. Without eavesdropping, the receiver recovers the point cloud semantic information x.

The semantic features y received by the decoder network are obtained by transmitting x through a quantum channel and used to obtain the reconstructed point cloud $P^{\prime }\in {\mathbb{R}}^{N\times 3}$. First, y is processed by 2 point restoration (PR) layers (each including a 1D transposed convolution [47]), which transform $y\in {\mathbb{R}}^{1\times n}$ into the point cloud feature $y_{0}\in {\mathbb{R}}^{N\times f}$ (*f* denotes the number of output feature channels). Then, y and $y_{0}$ are input into the point cloud feature restoration (PCFR) layers. The first PCFR layer takes $y_{0}$ as input, and subsequent layers use the previous layer’s output as input. The PCFR decoder is designed to address the core challenge of rebuilding high-dimensional, sparse 3D spatial information from a highly compressed semantic representation. The transmitted semantic vector y serves as a conditioning signal that guides feature refinement at each layer: y provides global semantic context that directs the allocation of point densities in the high-dimensional feature space, enabling the decoder to simultaneously preserve global structural integrity and recover local geometric details from the limited semantic code. The PCFR layers map features to different dimensions and guide semantic fusion through y, following the point condition network (PCNet) architecture [48]. The final output feature dimension of the PCFR layer is 3, resulting in the reconstructed point cloud $P^{\prime }\in {\mathbb{R}}^{N\times 3}$.

The system is trained end-to-end to optimize the reduction of the difference between the original point cloud distribution P and the reconstructed one $P^{\prime }$. CD [49], a commonly used metric, measures the difference between 2 point clouds by computing their bidirectional distance. It is computationally efficient and can capture the geometric discrepancy between the original and reconstructed point clouds, so it is suitable as both a loss function and an evaluation metric. The CD is defined as

$$\begin{array}{l} \mathrm{CD}\left(P,\;P^{\prime }\right)=\frac{1}{|P|}\sum_{a_{i}\in P}\underset{b_{j}\in P^{\prime }}{\text{min}}∥a_{i}-b_{j}{∥}_{2}^{2}\\ \;+\frac{1}{|P^{\prime }|}\sum_{b_{j}\in P^{\prime }}\underset{a_{i}\in P}{\text{min}}∥b_{j}-a_{i}{∥}_{2}^{2}. \end{array}$$ (1)

where $a_{i}$ and $b_{j}$ denote the *i*th and *j*th points in P and $P^{\prime }$, respectively, with i,j=1,2,…,N. A smaller CD value indicates higher reconstruction accuracy, and a CD of zero implies that the reconstructed point cloud is identical to the original.

### Semantic encoder

**Fig. 4.**
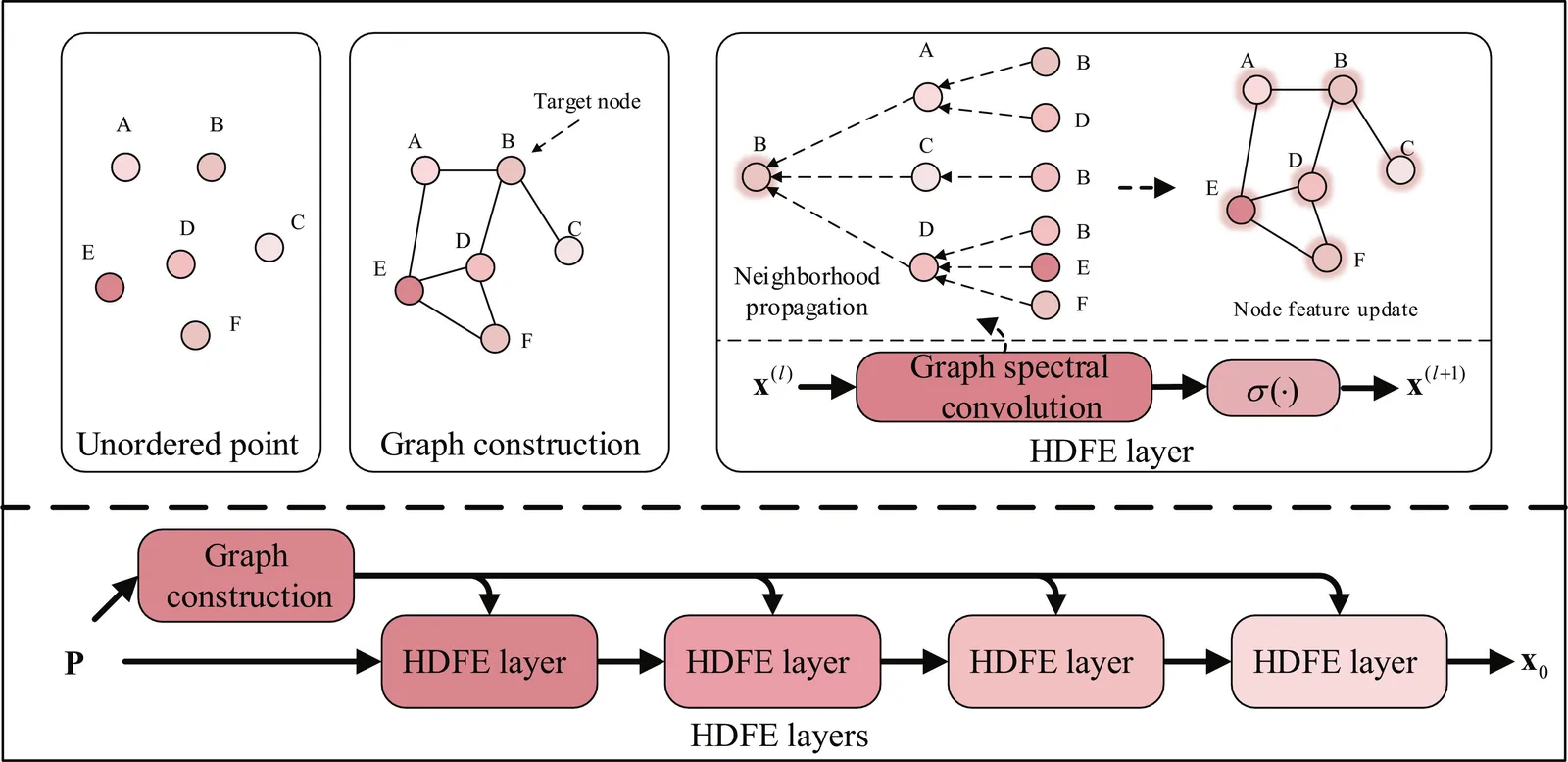
The framework of the HDFE layers.

The primary task in designing a semantic communication system is to extract the semantic features of the data. Considering that point clouds are unstructured, unordered, and discrete, we leverage the graph convolutional feature learning framework suitable for processing non-Euclidean data to capture the deep semantic features of point clouds. Graph convolution respects the inherent nature of point clouds as spatial point sets. It explicitly encodes 3D spatial relationships via dynamic local graph construction and enables efficient feature propagation and aggregation through message-passing mechanisms. The HDFE layer, constructed with graph convolution as its core component, is illustrated in Fig. 4. Specifically, for the input point cloud P, taking each point in the point cloud as a node of the graph, we select the *k*-NN for each point using the *k*-NN algorithm [45] to construct the undirected graph structure $G=\left(V,E\right)$ with *N* nodes $v_{i}\in V$, edges $\left(v_{i},\;v_{j}\right)\in E$, an binary adjacency matrix $A\in {\mathbb{R}}^{N\times N}$, and a degree matrix ${\tilde{D}}_{\mathrm{ii}}=\sum_{j}A_{\mathrm{ij}}$. Subsequently, the graph spectral convolution [46] is used to propagate and aggregate the features of each point according to the graph structure G, completing the update of node features, as shown on the upper side of Fig. 4. The calculation process is formulated as

$$x^{\left(l+1\right)}=\sigma \left({\tilde{D}}^{\frac{1}{2}}{\tilde{A}\tilde{D}}^{\frac{1}{2}}x^{\left(l\right)}\Theta^{\left(l\right)}\right),$$ (2)

where $\tilde{A}=A+I_{A}$ is the adjacency matrix of the undirected graph G with added self-connections. $I_{A}$ is the identity matrix, ${\tilde{D}}_{\mathrm{ii}}=\sum_{j}{\tilde{A}}_{\mathrm{ij}}$ and $\Theta^{\left(l\right)}$ is a layer-specific trainable weight matrix. $\sigma \left(\cdot \right)$ denotes an activation function. To capture rich hierarchical features of the point cloud, we design 4 HDFE layers to iteratively enlarge the receptive field of each point, and the final output $x_{0}$ is obtained as the aggregated multiscale feature representation of the entire point cloud.

Next, to obtain a global representation of multiscale features of the point cloud, we employ a linear layer to capture statistical information and project the channel dimension to the target channel dimension. This process can be formulated as

$$x_{0^{\prime }}=W_{f}x_{0}+b_{f},$$ (3)

where $W_{f}\in {\mathbb{R}}^{d\times n}$ is a learnable weight matrix of the fully connected (linear) layer, *n* denotes the target channel dimension, and $b_{f}$ is a bias vector of the linear layer. Subsequently, to reduce the volume of transmitted data, we perform channel-wise global max-pooling on $x_{0^{\prime }}\in {\mathbb{R}}^{N\times n}$ to obtain a global semantic representation $x_{1^{\prime }}\in {\mathbb{R}}^{1\times n}$. The specific calculation is

$${\left(x_{1^{\prime }}\right)}_{j}=\underset{i\in \left\{1,2,\ldots \;N\right\}}{\text{max}}{\left(x_{0^{\prime }}\right)}_{\mathrm{ij}},j=1,\ldots ,n.$$ (4)

Finally, to meet the power constraint, $x_{1^{\prime }}$ is fed into the power normalization layer to generate a codeword x. Specifically, power normalization is calculated using the global mean and standard deviation of $x_{1^{\prime }}$, i.e.,

$$x=\frac{x_{1^{\prime }}-\mu_{x}}{\sigma_{x}},$$ (5)

where $\mu_{x}$ denotes the global mean and $\sigma_{x}$ denotes the global standard deviation of $x_{1^{\prime }}$, and the normalized codeword x is then transmitted through the quantum channel.

### Semantic decoder

The semantic features $y\in {\mathbb{R}}^{1\times n}$ received by the semantic encoder are obtained by transmitting x through a quantum channel, and y is further used to reconstruct the point cloud $P^{\prime }\in {\mathbb{R}}^{N\times 3}$. To recover the number of points in the point cloud, we feed y into 2 sequential PR layers, which map y to $y_{0}\in {\mathbb{R}}^{N\times f}$ (restoring the original number of point cloud points) with each layer essentially a 1D deconvolution (Deconv) with a unit stride [47]. The 2-step calculation is as follows:

$$y_{0}=\text{Deconv}\left(\text{Deconv}\left(y\right)\right).$$ (6)

However, the obtained point cloud features $y_{0}$ are susceptible to noise interference introduced during quantum channel transmission, leading to inaccuracies in point density distribution. To address this issue, we further refine the features by leveraging the high-dimensional semantic information of y, inspired by the PCNet architecture [48]. Specifically, we use y to guide the density allocation of points in $y_{0}$ within the high-dimensional feature space.

**Fig. 5.**
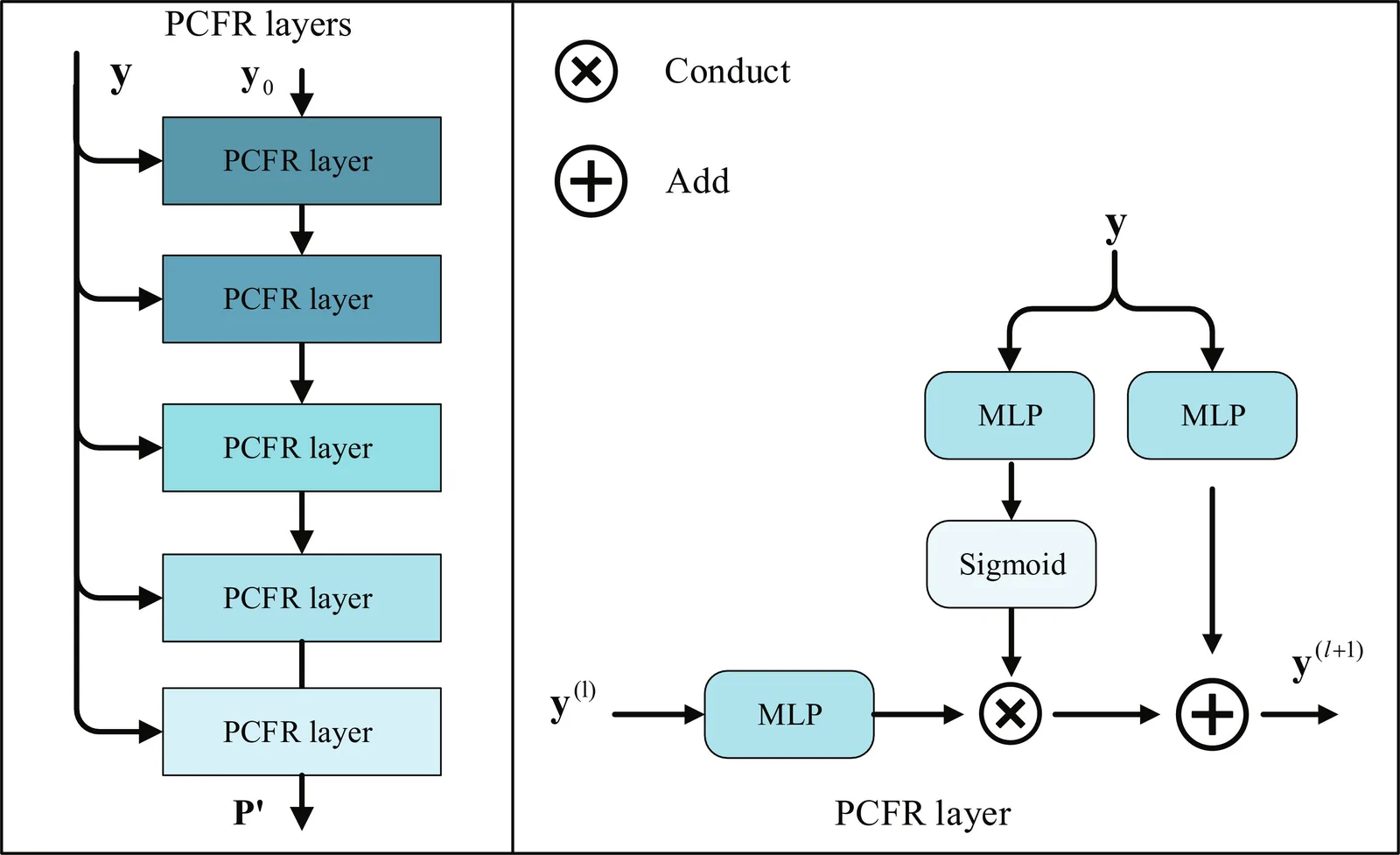
The framework of the PCFR layers.

To implement this feature refinement, we adopt 5 stacked FCFR layers, and the structural details of FCFR layer (illustrated in Fig. 5) are designed to incorporate the semantic guidance of y at each layer, where we denote the feature output of the *l*th FCFR layer as $y^{\left(l+1\right)}$. The mathematical formulation of each FCFR layer is given by

$$y^{\left(l+1\right)}=F_{l}⊙\left(W_{\mathrm{yh}}^{\left(l\right)}y^{\left(l\right)}+b_{\mathrm{yh}}^{\left(l\right)}\right)+W_{\mathrm{yb}}^{\left(l\right)}y,$$ (7)

where $F_{l}=\sigma \left(W_{\mathrm{yl}}^{\left(l\right)}y+b_{\mathrm{yl}}^{\left(l\right)}\right)$, σ represents the sigmoid function, $y^{\left(0\right)}=y_{0}$, and $W_{y*}^{\left(l\right)},b_{y*}^{\left(l\right)}$ are all trainable parameters. As the point cloud features pass through the FCFR layers sequentially, they first undergo dimensionality expansion to enrich feature representation, followed by noise filtering to mitigate the impact of transmission noise. Subsequently, the features are subjected to dimensionality reduction, and finally restored to the original dimension of the point cloud, thereby achieving the reconstruction of point cloud $P^{\prime }\in {\mathbb{R}}^{N\times 3}$.

### STIKE one-way quasi-QSDC experiment

**Fig. 6.**
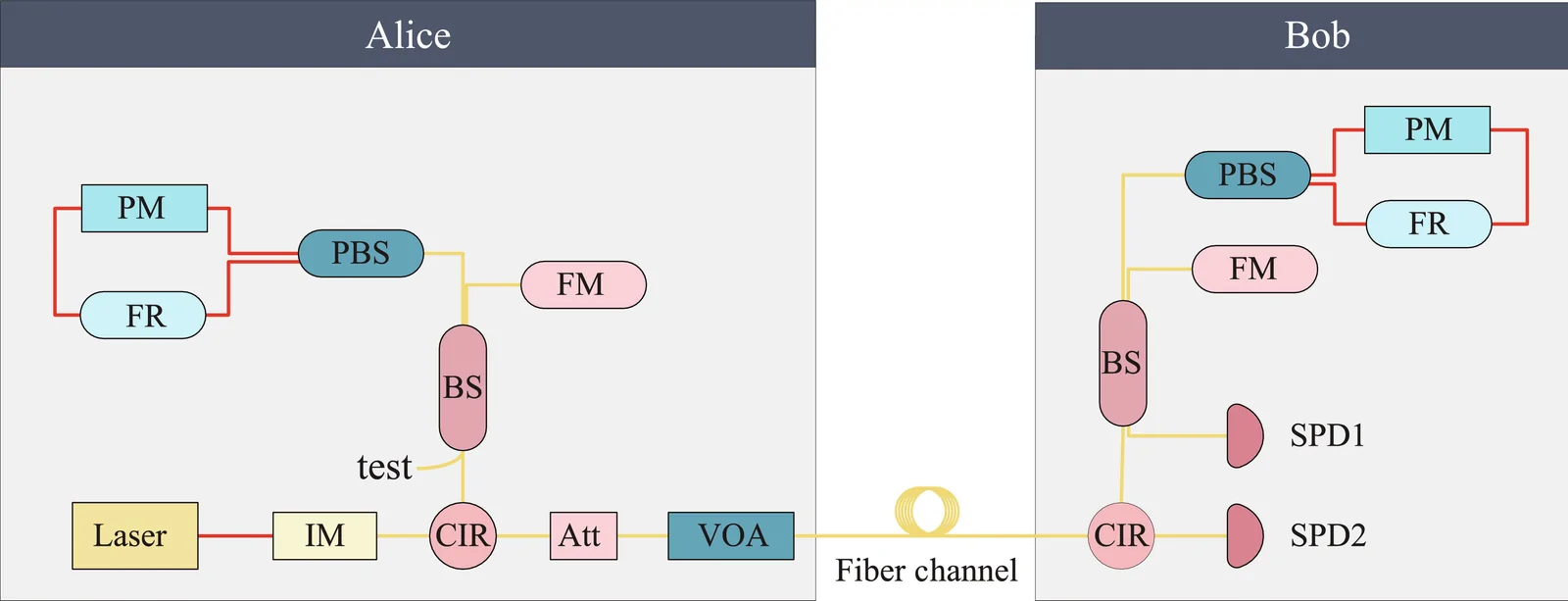
Experimental setup for the one-way quasi-QSDC protocol. Att, attenuator; BS, beam splitter; CIR, circulator; FM, Faraday mirror; FR, Faraday rotator; IM, intensity modulator; PBS, polarization beam splitter; PM, phase modulator; SPD, single-photon detector; VOA, variable optical attenuator.

In the experiment, the one-way quasi-QSDC protocol using single photons has been applied for secure and reliable transmission [25]. It preserves the transmission of information via quantum states, while still relying on a preshared key and classical one-time-pad encryption for security. First, the quantum transmitter named Alice encodes the semantic information using forward error correction and encrypts it with a shared secure key via a one-time pad, then further masks the ciphertext using a local random sequence. She converts the masked data into quantum states by encoding bits into randomly chosen measurement bases and sends the photons to the quantum receiver named Bob. Bob randomly measures the incoming photons, records successful detections, and publicly announces timing and basis information so both parties can keep only the matched-basis results and estimate the error rate to detect eavesdropping. Alice then reveals the masking randomness for the retained events, allowing Bob to recover a noisy ciphertext, which he decrypts using the shared key and corrects using error correction to obtain the original semantic message. Finally, keys corresponding to lost photons are recycled, while additional fresh secure keys are generated through standard quantum key distribution postprocessing, maintaining the key supply for future communication. As shown in Fig. 6, Faraday–Michelson systems have been employed for phase encoding and decoding.

### Secrecy channel capacity

We consider a one-way QSDC configuration over a transmission distance *L*. The overall detection probability is determined by the effective channel efficiency η, which is written as

$$\eta =10^{-\alpha L/10}\;t_{\mathrm{Bob}}\;\eta_{D}.$$ (8)

Here, α=0.2dB/km denotes the fiber attenuation coefficient. The factor $t_{\mathrm{Bob}}$ accounts for the internal transmittance at Bob’s receiver, and $\eta_{D}$ is the detector efficiency.

For a signal state with mean photon number μ, the total gain is given by

$$Q_{\mu }=Y_{0}+1-e^{-\eta \mu }.$$ (9)

The corresponding QBER reads

$$E_{\mu }=\frac{e_{0}Y_{0}+e_{\text{det}}\left(1-e^{-\eta \mu }\right)}{Q_{\mu }}.$$ (10)

The background yield is $Y_{0}=2p_{d}$, which arises from detector dark counts. The parameter $e_{0}=1/2$ represents the error probability of background events, while $e_{\det}$ characterizes intrinsic detector imperfections.

To bound the single-photon contribution, we apply a 2-decoy-state scheme. Two decoy intensities are used, $\nu_{1}=\nu$ and $\nu_{2}=0$. Under this choice, the gain of the decoy state is

$$Q_{\nu }=Y_{0}+1-e^{-\eta \nu }.$$ (11)

The lower bound of the single-photon fraction $\Delta_{1}$ is obtained as

$$\Delta_{1}\geq \frac{\mu^{2}e^{-\mu }}{\mu \nu -\nu^{2}}\left(\frac{Q_{\nu }}{Q_{\mu }}e^{\nu }-\frac{\nu^{2}}{\mu^{2}}e^{\mu }-\frac{\mu^{2}-\nu^{2}}{\mu^{2}}\frac{Y_{0}}{Q_{\mu }}\right).$$ (12)

The single-photon error rate is upper bounded by

$$e_{1}\leq \frac{E_{\nu }Q_{\nu }e^{\nu }-e_{0}Y_{0}}{Y_{1}^{L,\nu ,0}\nu },$$ (13)

where $E_{\nu }$ denotes the QBER of the decoy state,

$$E_{\nu }=\frac{e_{0}Y_{0}+e_{\text{det}}\left(1-e^{-\eta \nu }\right)}{Q_{\nu }}.$$ (14)

The corresponding lower bound of the single-photon yield is given by

$$Y_{1}^{L,\nu ,0}=\frac{\mu }{\mu \nu -\nu^{2}}\left(Q_{\nu }e^{\nu }-Q_{\mu }e^{\mu }\frac{\nu^{2}}{\mu^{2}}-\frac{\mu^{2}-\nu^{2}}{\mu^{2}}Y_{0}\right).$$ (15)

Based on the above quantities, the secrecy capacity of the QSDC system derived from the Shannon–Wyner entropy is defined as

$$C_{\mathrm{SW}}=\frac{1}{2}Q_{\mu }\left\{-{\mathrm{fH}}_{2}\left(E_{\mu }\right)+\Delta_{1}\left[1-H_{2}\left(e_{1}\right)\right]\right\},$$ (16)

which accounts for both the information leakage due to error correction and the secure contribution from single-photon states. Here, the first term represents the classical error-correction cost where *f* denotes the error correction efficiency, and the second term quantifies the extractable secure information against eavesdropping.

For comparison, the Shannon capacity characterizes the maximum rate of reliable information transmission between the quantum transmitter and quantum receiver. It can be written as

$$C_{S}=\frac{1}{2}Q_{\mu }\left[1-{\mathrm{fH}}_{2}\left(E_{\mu }\right)\right],$$ (17)

which corresponds to the mutual information shared by the legitimate parties in the presence of channel errors. By substituting parameters in Table 2 into Eqs. (16) and (17), the corresponding values shown in Fig. 2E can be obtained.

**Table 2.** Experimental parameters of the S-QSDC-PC system

Parameter	Annotation	Value
*α*	Loss coefficient of the fiber channel	0.2 dB/km
*e*_det_	Intrinsic detector error rate	1.3%
*η*_D_	Detector efficiency	20%
*f*	Error correction efficiency	1.05
*L*	Transmission distance of single photons	50 km
*μ*	The mean photon number of the signal state	0.6
*v*	The mean photon number of the decoy state	0.2
*P*_d_	Dark count rate of single-photon detector	1.2 × 10^−6^
*t*_Bob_	The internal transmittance of Bob’s end	10^−6.5/10^

## Data Availability

Data are available from the corresponding authors upon reasonable request.
